# The availability of the embryonic TGF-β protein Nodal is dynamically regulated during glioblastoma multiforme tumorigenesis

**DOI:** 10.1186/s12935-016-0324-3

**Published:** 2016-06-17

**Authors:** Maria Cecília Oliveira-Nunes, Suzana Assad Kahn, Ana Luiza de Oliveira Barbeitas, Tania Cristina Leite de Sampaio e Spohr, Luiz Gustavo Feijó Dubois, Grasiella Maria Ventura Matioszek, William Querido, Loraine Campanati, José Marques de Brito Neto, Flavia Regina Souza Lima, Vivaldo Moura-Neto, Katia Carneiro

**Affiliations:** Institute of Biomedical Sciences, Federal University of Rio de Janeiro, Av. Carlos Chagas Filho 373, F2-01, Rio de Janeiro, Rio de Janeiro 21941-902 Brazil; Institute for Stem Cell Biology and Regenerative Medicine, Stanford University, 265 Campus Drive, Stanford, California 94305 USA; Institute of Biomedical Sciences, Federal University of Rio de Janeiro, Av. Carlos Chagas Filho 373, F1-20, Rio de Janeiro, Rio de Janeiro 21941-902 Brazil; Instituto Estadual do Cérebro Paulo Niemeyer (IECPN), Rua do Rezende, 156, Rio de Janeiro, Rio de Janeiro 20231-092 Brazil; Institute of Biomedical Sciences, Federal University of Rio de Janeiro, Av. Carlos Chagas Filho 373, B1-29, Rio de Janeiro, Rio de Janeiro 21941-902 Brazil; Institute of Biomedical Sciences, Federal University of Rio de Janeiro, Av. Carlos Chagas Filho 373, F2-30, Rio de Janeiro, Rio de Janeiro 21941-902 Brazil

**Keywords:** Glioblastoma, Cancer stem-cell, Nodal, Endocytosis, Tumorigenesis

## Abstract

**Background:**

Glioblastoma (GBM) is the most common primary brain tumor presenting self-renewing cancer stem cells. The role of these cells on the development of the tumors has been proposed to recapitulate programs from embryogenesis. Recently, the embryonic transforming growth factor-β (TGF-β) protein Nodal has been shown to be reactivated upon tumor development; however, its availability in GBM cells has not been addressed so far. In this study, we investigated by an original approach the mechanisms that dynamically control both intra and extracellular Nodal availability during GBM tumorigenesis.

**Methods:**

We characterized the dynamics of Nodal availability in both stem and more differentiated GBM cells through morphological analysis, immunofluorescence of Nodal protein and of early (EEA1 and Rab5) and late (Rab7 and Rab11) endocytic markers and Western Blot. Tukey’s test was used to analyze the prevalent correlation of Nodal with different endocytic markers inside specific differentiation states, and Sidak’s multiple comparisons test was used to compare the prevalence of Nodal/endocytic markers co-localization between two differentiation states of GBM cells. Paired t test was used to analyze the abundance of Nodal protein, in extra and intracellular media.

**Results:**

The cytoplasmic distribution of Nodal was dynamically regulated and strongly correlated with the differentiation status of GBM cells. While Nodal-positive vesicle-like particles were symmetrically distributed in GBM stem cells (GBMsc), they presented asymmetric perinuclear localization in more differentiated GBM cells (mdGBM). Strikingly, when subjected to dedifferentiation, the distribution of Nodal in mdGBM shifted to a symmetric pattern. Moreover, the availability of both intracellular and secreted Nodal were downregulated upon GBMsc differentiation, with cells becoming elongated, negative for Nodal and positive for Nestin. Interestingly, the co-localization of Nodal with endosomal vesicles also depended on the differentiation status of the cells, with Nodal seen more packed in EEA1/Rab5 + vesicles in GBMsc and more in Rab7/11 + vesicles in mdGBM.

**Conclusions:**

Our results show for the first time that Nodal availability relates to GBM cell differentiation status and that it is dynamically regulated by an endocytic pathway during GBM tumorigenesis, shedding new light on molecular pathways that might emerge as putative targets for Nodal signaling in GBM therapy.

**Electronic supplementary material:**

The online version of this article (doi:10.1186/s12935-016-0324-3) contains supplementary material, which is available to authorized users.

## Background

Nodal is a member of the TGF-β superfamily of secreted proteins that signals through the serine/threonine kinase receptors family triggering the phosphorylation of Smads 2 and 3 [1]. Among a range of biological functions attributed to Nodal protein is its classical role during embryonic development [1–3], stem cell development and differentiation [4]. However, recent studies have shown that Nodal also regulates the maintenance of pluripotency in embryonic stem cells [5], carcinogenesis [6], and tumor cell progression and development [7–15].

Glioblastoma (GBM; grade IV astrocytoma) is the most common primary brain tumor characterized by aggressive invasiveness, high proliferative rate, insensitivity to radio- and chemotherapy and a short survival period [16–18]. It has been reported that the tumoral mass is generated by a rare fraction of cells displaying self-renew capacity named tumor-initiating cells [19–22] that are involved in tumor growth and resistance to chemotherapy [23]. Sub-population of GBM cells with stem-like properties may be the source of tumors since, apparently, these stem cells are highly resistant to current cancer treatments and survive to regenerate new tumors [16, 24, 25].

Regardless the physiological function of Nodal has been extensively described elsewhere during tumor progression and tumorigenesis, the characterization of Nodal availability in this context has not been addressed so far. In this study we have investigated by an original approach the dynamics of Nodal intracellular distribution and extracellular availability in both stem and more differentiated GBM cells. Strikingly, we found that in GBM stem cells Nodal co-localizes in early endosomal vesicles and is abundantly available both intra and extracellularly. On the other hand, Nodal was found to co-localize to late endosomal compartments, including lysosomal vesicles, and was less available in the extracellular medium in more differentiated GBM cells. Altogether, our results propose for the first time that the Nodal availability is controlled by an endocytic pathway during GBM tumorigenesis shedding light on the molecular pathways that might emerge as putative targets for GBM therapy.

## Methods

### Cell culture

The cells were cultured as described in [26]. The human glioblastoma cell line GBM011 (mdGBM) [27] and OB1 stem cells (GBMsc) [28] were obtained in previous studies. The human GBM U87MG cell line (mdGBM) was purchase from ATCC (Manassas, VA, USA). The experiments with human cells were regulated by the license MS, CONEP 2340. DU145 prostate cell line was purchased from the Cell Bank of Rio de Janeiro—UFRJ.

### Immunocytochemistry

Permeabilization was done with 3 % Triton X-100 in PBS followed by 10 % BSA in PBS incubation. Primary anti-Nodal (1:25, rabbit, H-110, Santa Cruz Antibodies), anti-Nestin (1:200, Promega), anti-EEA1 (1:100, Santa Cruz sc5939), anti-Rab5 (1:100, Santa Cruz sc46692), Rab7 (1:100, Santa Cruz Antibodies sc6563) and Rab11 (1:100, Santa Cruz sc9020) were used followed by secondary antibody incubation (1:300, Molecular Probes) plus DAPI nuclear stain (1 μg/1 μl). The slides for Nodal immunostaining were incubated with tyramide (1:100, TSA kit #13, Life Technologies, T-20923) and were mounted with Vectashield (Vector Laboratories). Leica TCS SP5 AOBS confocal microscope was used. The images were handled in Image J. Intensity and co-localization analysis was perfomed by Leica Application Suite (LAS), Leica Microsystems and Pearson’s correlation coefficient (PCC) was used for statistic quantifying colocalization. We analyzed three different fields for each marker, where we performed the co-localization analysis in a mean of three spheroids or 20–30 cells per field. The prevalence of Nodal colocalization with different endocytic markers in each cell line was analyzed by Tukey’s test. For comparison and analysis of Nodal/endocytic markers between two cell lines, we used Sidak’s multiple comparisons test.

### Western blot

RIPA buffer solution was used in the presence of protease inhibitors. The protein samples were separated by electrophoresis and blotted to PVDF membrane, followed by blocking with 5 % non-fate dry milk powder in 0.1 % PBS-Tween and incubated overnight with primary antibody for Nodal. The secondary antibody used was the same for Nodal immunofluorescence assays. The reaction was developed using SuperSignal^®^ West Pico Chemoluminescent Substrate (Thermo Scientific) and gray scale analysis of protein bands was performed using image software. Loading control and normalization was performed through α-tubulin and actin immunobloting, and quantifications were performed in Image J.

### Conditioned medium acquirement

OB1 stem cell and OB1 cells subjected to differentiation were cultivated with DMEM in the absence of Fetal Serum Bovine (FSB) for 3 days to acquire Conditioned Medium (CM). Proteins present in the CM were precipitated with 80 % acetone at −20 °C overnight and the protein extracts were processed by Western Blot assays as described above. Here, loading control and normalization was performed through densitometry of Coomassie Blue R staining of the gel. Quantifications handled on Image J.

### Statistical analysis

GraphPad Prism (v6.0, La Jolla, CA) was used for ordinary *one*-*way* or *two*-*way* ANOVA analysis where appropriate. If the ANOVA produced a significant result, post hoc pair-wise comparisons were tested for significance in which the *P* value was adjusted (*P* adj < 0.05) by the Tukey’s method for multiple comparisons inside each group and by the Sidak’s method for multiple comparisons among the individual groups. Results are presented as mean ± SD and statistical relevance was defined as *P* < 0.05.

## Results

### Nodal protein intracellular distribution depends on GBM differentitation status

**Fig. 1:**
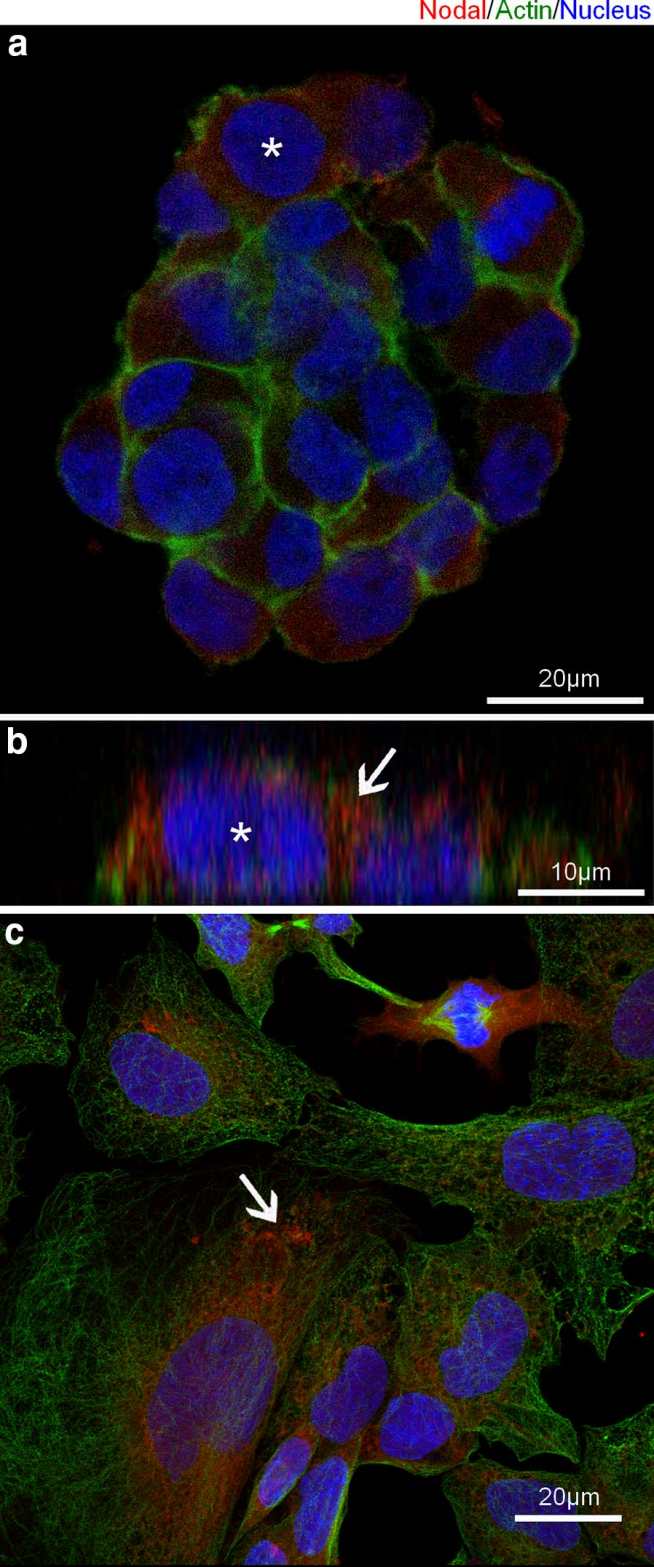
Nodal protein intracellular distribution depends on GBM differentitation status. **a** Nodal immunostaining was symmetrically distributed in the cytoplasm of OB1 stem cells. **b** Optical slices projected on the YZ axis showing a virtual reconstruction of the cell seen in “a” (*asterisk*). Nodal was localized to vesicle-like particles (*arrow*). **c** In GBM011 cells, Nodal immunostaining was found in a rare population of cells and was asymmetrically localized to a perinuclear region. Vesicle-like particle were also observed (*arrow*)

Nodal immunostaining was detected symmetrically distributed in the cytoplasm of OB1 stem cells (Fig. 1a), appearing as vesicle-like subcellular particles (Fig. 1b, arrow). Additionally, we noticed that the presence of Nodal in these cells changed along the development of the oncospheres. While small spheres contained only cells that show a symmetrical Nodal distribution (Additional file 1: Figure S1a), large ones were comprised by cells with distinct Nodal distributions (Additional file 1: Figure S1b). Nodal-positive cells were found on top of the oncospheres and presented Nodal immunostaining mainly localized to points of cell–cell contact (Additional file 1: Figure S1c–e). Nodal immunostaining was detected symmetrically distributed in the cytoplasm of cells located on the lateral edges, surrounding the whole oncosphere (Additional file 1: Figure S1f–h), as well as in the cytoplasm of cells that were directly attached to the substrate (Additional file 1: Figure S1i–l). The lack of the initially seen Nodal protein symmetrical distribution in some cells was most likely was due to the beginning of their differentiation in the interior of the oncospheres. Thus we conclude that Nodal is localized to vesicles-like particles that are symmetrically distributed in the cytoplasm of undifferentiated OB1 stem cells.

In GBM011 cells, Nodal immunostaining was present in an asymmetric pattern, characterized by a perinuclear distribution, with no evidence of association to the plasma membrane (Fig. 1c). Nodal positive vesicle-like particles were observed, and unlike OB1 stem cells, presented a perinuclear localization (Fig. 1c, arrow).

To test whether Nodal asymmetric distribution is restricted to mdGBM cells or if it is a broader marker of differentiated cancer cells, we also tested and found the same asymmetry in the well-described prostate cell line DU145 that has been shown to transduce the Nodal signaling pathway (Additional file 2: Figure S2). Thus we conclude that Nodal immunostaining is found in asymmetric perinuclear localization in more differentiated GBM cells during interphase.

### Nodal asymmetric cytoplasmic distribution shifts to a symmetric distribution in dedifferentiated GBM cells

We next tested whether the Nodal asymmetric and symmetric distributions represent markers of the differentiation state of GBM cells. We reasoned that the asymmetric distribution, found in mdGBM cells, would shift to a symmetric distribution, if these cells were induced to a less differentiated status. To test this hypothesis, we have used the U87MG cell line induced with a dedifferentiation protocol. We verified the differentiation status through Nanog Western Blot, in OB1, differentiated OB1, U87MG and U87MG dedifferentiated cells (Additional file 3: Figure S3). In the original differentiated cells, Nodal immunostaining was asymmetrically located in the perinuclear area (Additional file 4: Figure S4, arrows). Conversely, when U87MG cells were induced to dedifferentiate (U87MG-O), the Nodal asymmetric distribution shifted to a symmetric pattern (Additional file 4: Figure S4b–d, arrows), similar to that observed in OB1 stem cells. We also found that U87MG-O cells upregulate Nodal protein intracellular levels, reinforcing our data obtained on OB1 cells. This finding indicates that Nodal cytoplasmic distribution and levels are dynamically regulated and strongly correlate with the differentiation status of GBM cells.

### Nodal protein intra and extracellular levels are reduced upon GBM differentiation

**Fig. 2:**
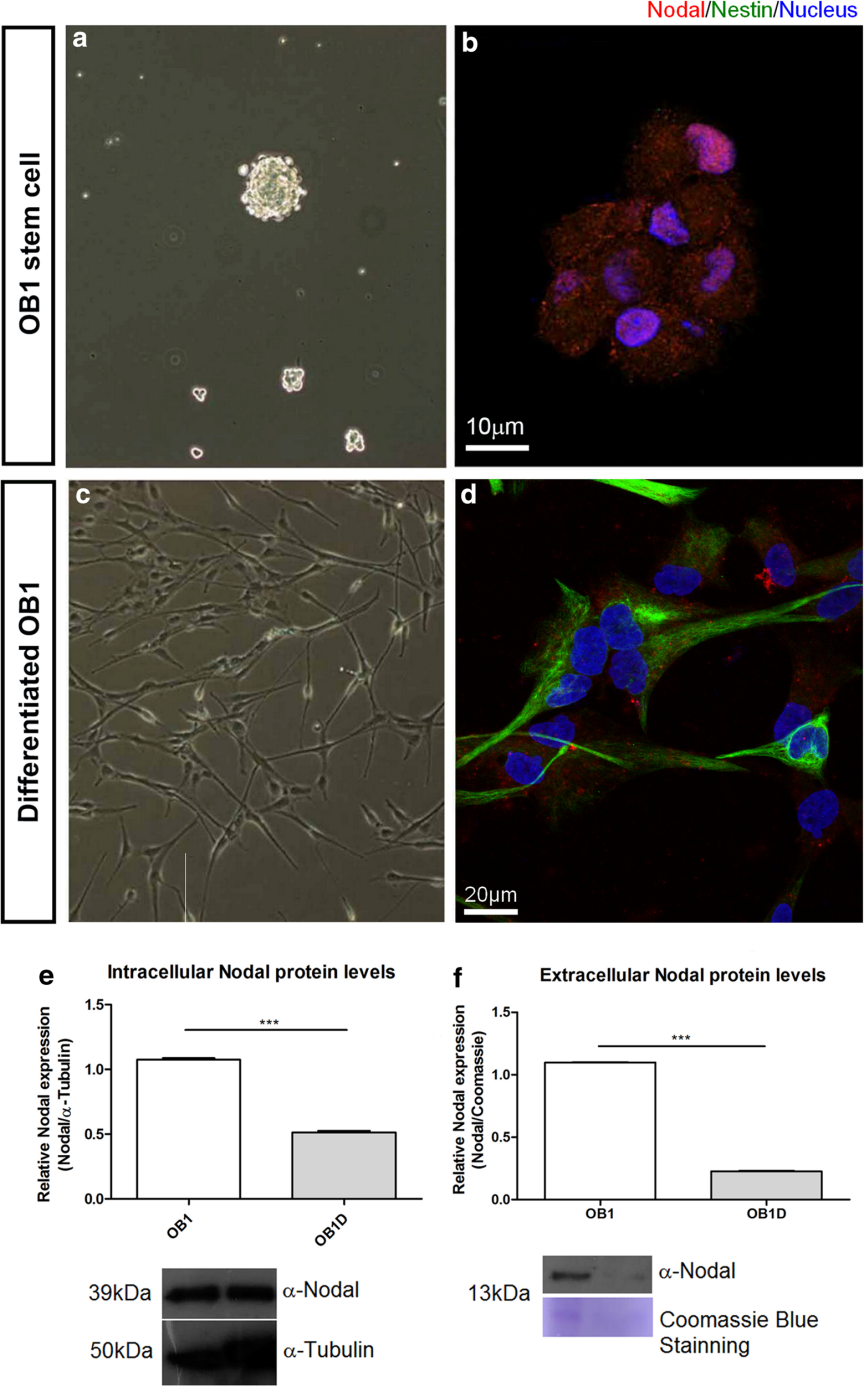
Nodal protein levels are dowregulated during GBM differentiation. **a**, **b** OB1 stem cells forming oncospheres were immunostained for Nodal (*red*) and Nestin (*green*). DAPI (*blue*). OB1 stem cells present Nodal immunostaining localized to vesicles-like particles symmetrically distributed in the cytoplasm of GBM stem cells that are Nestin-negative (**c**, **d**). Upon differentiation, less differentiated cells were found positive for both Nodal and Nestin. More differentiated GBM cells were found negative for Nodal and positive for Nestin (69 % of cells; **d**, *asterisk*). Cells presenting a less elongated morphology were found Nodal-positive and Nestin-negative (26 % of cells). **e** Quantification of intracellular Nodal protein in OB1 stem cells and upon differentiation by Western blot. Nodal protein levels decrease in 50 % upon differentiation (quantification of average across three separate experiments). Nodal protein normalization through α-Tubulin immunoblotting. **f** Quantification of extracellular Nodal protein in protein precipitation of proteins present in conditioned medium by Western blot, presenting visible decrease in Nodal levels in OB1 cells upon differentiation. Nodal protein normalization through Coomassie Blue staining of gel. Data are mean ± SD. ***P < 0.001 by unpaired t test, n = 3)

To better understand the dynamics of Nodal protein during the transition between a stem cell like to a more differentiated cell behavior of GBM cells, we have quantified the abundance of intracellular Nodal in OB1 stem cells induced to differentiate. To confirm the differentiation of the cells, we evaluated cell morphology and the distribution of Nestin, a marker for differentiating progenitor cells [29]. OB1 stem cells grown as spheres, in the absence of FBS, were Nestin-negative and Nodal positive (Fig. 2a, b). Differentiated OB1 cells presented a spread out morphology, were Nestin-positive and mostly negative for Nodal (69 %, n = 66 cells; Fig. 2c, d). As expected, in OB1 stem cells subjected to differentiation there was a decreased in Nodal protein levels (Fig. 2e; n = 3).

As Nodal is a member of the TGFβ superfamily, we asked whether the extracellular levels of Nodal are also dynamically regulated during GBM tumorigenesis. Protein quantification of the conditioned medium showed that Nodal extracellular levels were downregulated as GBMsc were induced to differentiate (Fig. 2f). These results show that not only Nodal distribution is altered but also its intra and extracellular availability are reduced upon GBMsc differentiation.

### Nodal protein co-localizes with different endosomal vesicles depending on the differentiation status of GBM cells

**Fig. 3:**
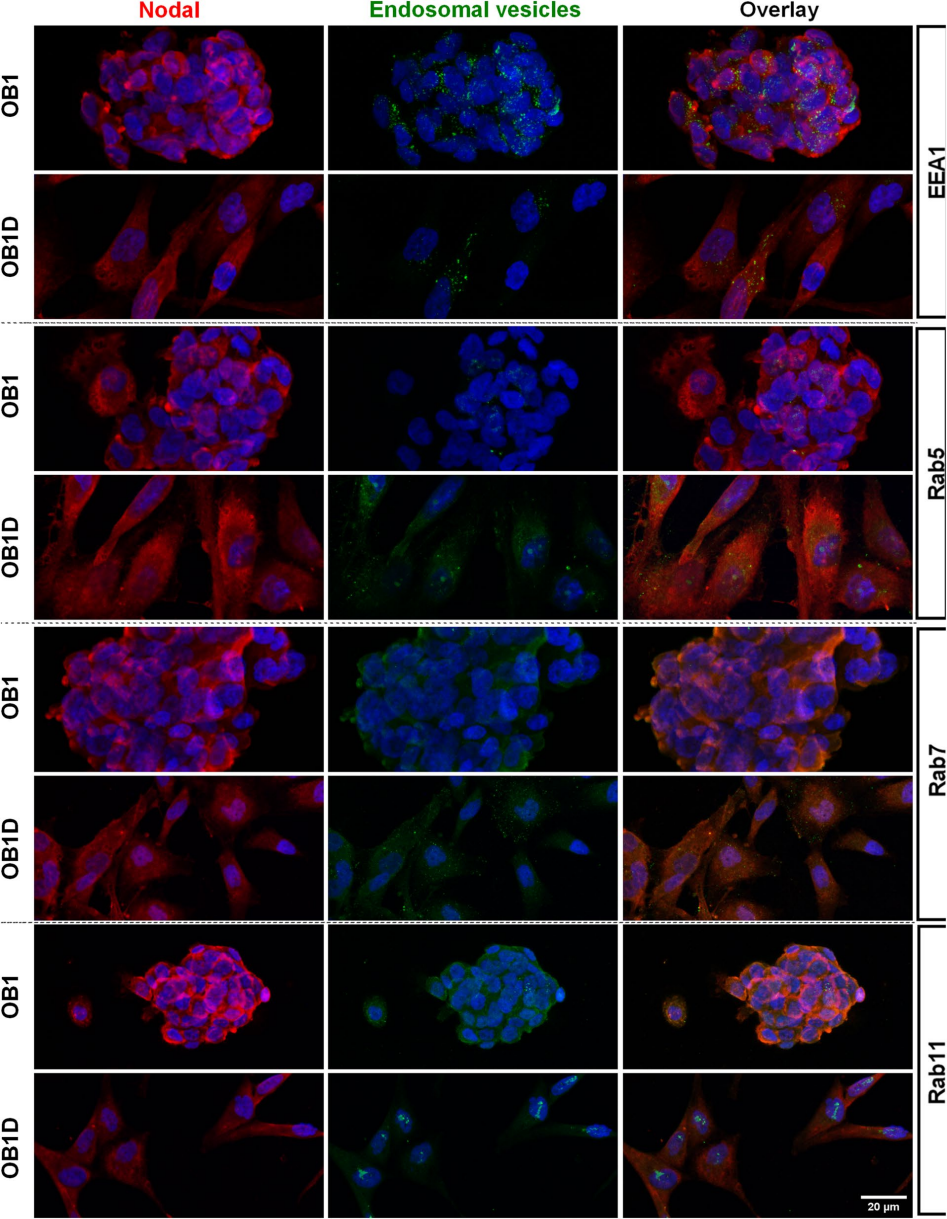
Nodal protein co-localizes with different endosomal vesicles depending on the differentiation status of GBM cells. Representative images of Nodal immunostaining with endosomal markers. In OB1 stem cells, Nodal co-localized with both early (EEA1 and Rab5) and late (Rab7 and Rab11) endosomes. In contrast, in differentiated OB1 cells, Nodal immunostaining was mostly co-localized with late (Rab7 and Rab11) endosomal vesicles

**Fig. 4:**
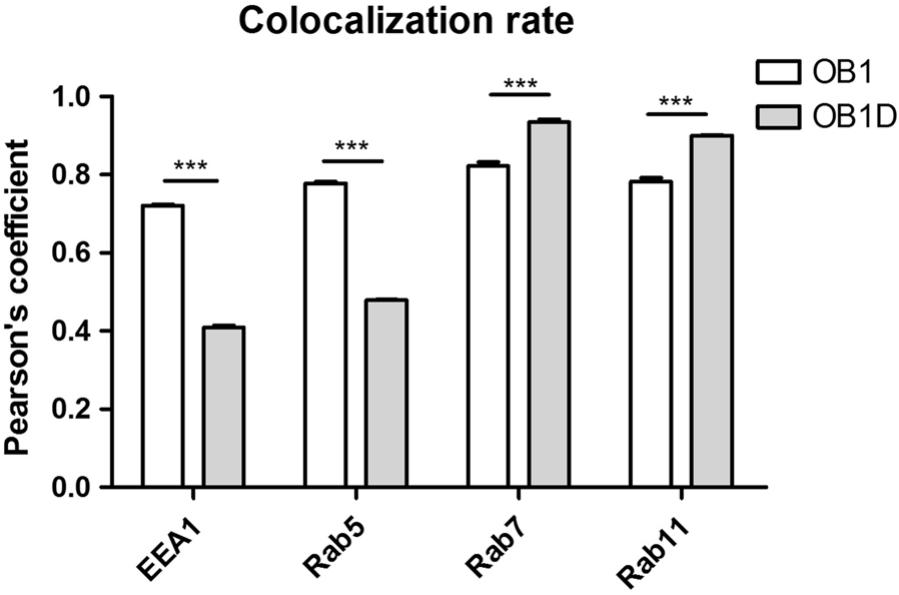
Nodal mostly co-localizes to Rab7 and Rab11 in more differentiated GBM cells. Pearson’s coefficient of relative amount of co-localization of endosomal markers/Nodal in OB1 and differentiated OB1 cell cultures (quantification of average across three separate fields, each containing an average of three to four spheroids—OB1 cells—or 20–30 cells—differentiated OB1 cells). Data means are ± SD. ***P < 0.001 by two-way ANOVA for repeated measures followed by Tukey’s test for correction of the *P* value

Previous studies have shown that Nodal was found in endosomal vesicles [30]. Therefore, to elucidate whether the vesicle-like particles observed in stem and differentiated GBM cells correspond to the same subcellular compartments or not or even whether they may change according to the differentiation status of GBM cells, we performed immuno co-localization of Nodal and different endosomal proteins in OB1 and differentiated OB1 cells (Figs. 3, 4). Nodal immunostaining in OB1 stem cells was found to co-localize with both early (EEA1 and Rab5) and late (Rab7 and Rab11) endosomes (Figs. 3, 4), suggesting that OB1 stem cells are continuously recycling and degrading Nodal. In contrast, in differentiated OB1 cells, Nodal immunostaining was mostly co-localized with late (Rab7 and Rab11) endosomal vesicles (Figs. 3, 4). These results are in agreement with a decrease in the levels of secreted Nodal in mdGBM cells as shown in Fig. 2f. It was possible to verify the same pattern and rates on U87MG and U87MG-O cells (Additional file 5: Figure S5, Additional file 6: Figure S6), suggesting that the Nodal endocytic processing is not specific of a cell line, relating to the phenotypic state. Also, our analysis of Nodal/endosomal markers co-localization in GBM011 cells reveal that the GBM primary cultures reproduced the pattern observed in mdGBM cells (Additional file 6: Figure S6). Thus, we conclude that Nodal availability might be controlled by an endocytic pathway, in a differentiation status dependent manner.

**Fig. 5:**
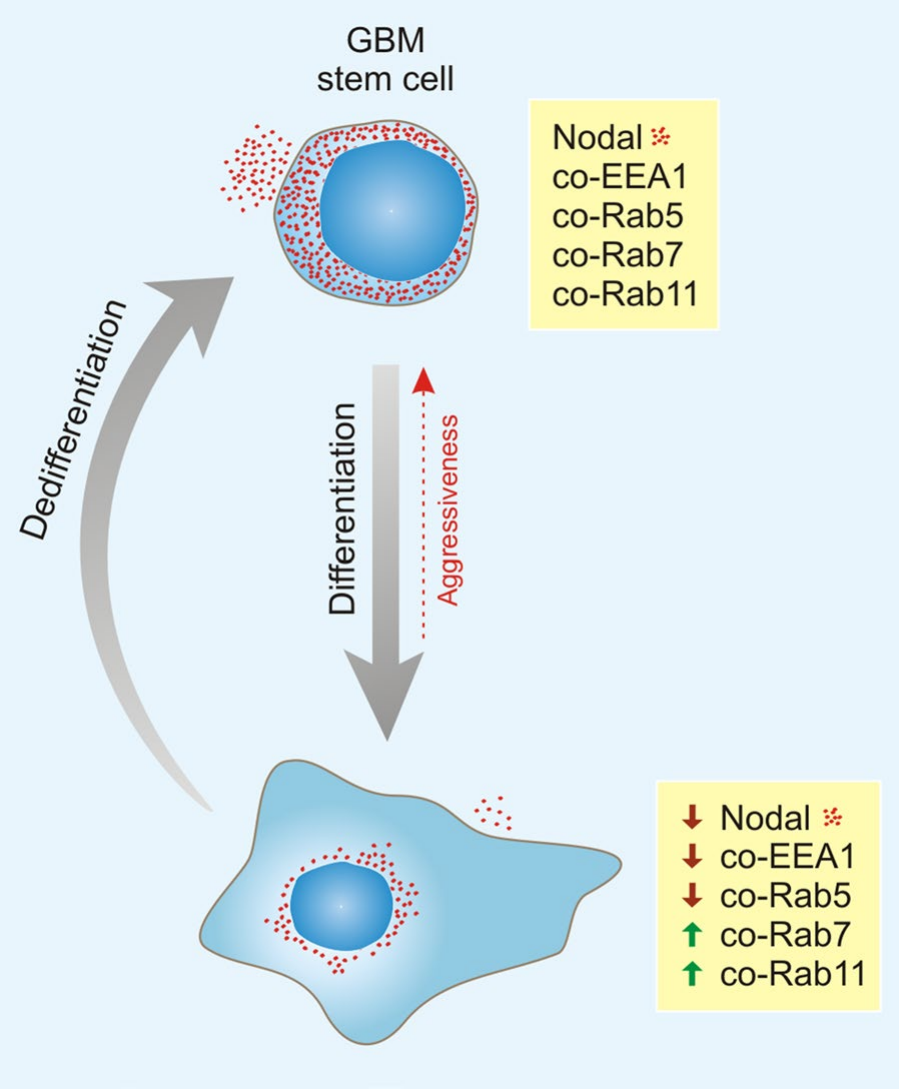
Illustration of the dynamics of Nodal distribution and availability during differentiation of GBM stem cells and of the endocytic mechanisms that may regulate Nodal during GBM tumorigenesis. The GBM stem cells shows a large amount of Nodal symmetrically distributed in their cytoplasm. The presence of Nodal in these cells is co-localization with both early (EEA1 and Rab5) and late (Rab7 and Rab11) endosomes. Upon differentiation, an asymmetric distribution of Nodal is found in the perinuclear region of the cells. In these cells, the intra and extracellular levels of Nodal are reduced and its co-localization with endosomes changes. There is a decrease in the association of Nodal with early (EEA1 and Rab5) endosomes and increase in its association with late (Rab7 and Rab11) endosomes. The characteristics seen in the stem cells can be returned after the dedifferentiation of the cells

Based on the results obtained in this study, it is possible to summarize in an illustration the dynamics of Nodal distribution and availability during differentiation of GBM stem cells, as well as the endocytic mechanisms that may regulate Nodal during GBM tumorigenesis (Fig. 5). Briefly, the GBM stem cells present a large amount of Nodal symmetrically distributed in their cytoplasm and presenting similar co-localization with both early (EEA1 and Rab5) and late (Rab7 and Rab11) endosomal vesicles. After differentiation, an asymmetric distribution of Nodal is seen in the cytoplasm, mostly limited to the perinuclear region. Moreover, the levels of Nodal in the cells are reduced and its co-localization with endosomal vesicles changes, showing a decrease in its association with early (EEA1 and Rab5) endosomes and increase in its association with late (Rab7 and Rab11) endosomes. The dedifferentiation of the cells can return these characteristics back those seen in the stem cells.

## Discussion

We provide novel data regarding Nodal protein dynamics during GBM tumorigenesis, a process that remains poorly characterized. Besides Nodal has already been shown as a substantial engine disposed by different types of cancer, its availability and dynamics has not been addressed so far. Since we consider the understanding of basic regulation of cancer machinery a fundamental factor for its approach, our study quest a better understanding of Nodal protein in a subcellular level on GBM. Using an original approach, we analyzed the dynamics of Nodal distribution and availability in GBM cells with distinct differentiation status. Through detailed subcellular immunofluorescence analysis, we showed for the first time that Nodal is dynamically regulated during GBM cell differentiation, with clear differences found between the stem and the more differentiated cells. We were able to observe not only that the Nodal cytoplasmic distribution varied between GBMsc and mdGBM, but also to confirm that it is strongly dependent upon the differentiation status to which each cell type is induced. Even more interestingly, we showed that the availability of Nodal is regulated by different endocytic pathways during GBM tumorigenesis, being associated with distinct endosomal vesicles depending on the differentiation status of the cells.

Vesicular intracellular trafficking has important roles in cell signaling coupling processing and endocytosis in signal-receiving cells [31, 32]. Endosomal markers are restrained at specific subcellular compartments, which dictates the stage of endosomal maturation of the vesicle [32]. Rab5 proteins are localized in early endosomes, which play essential roles in endocytosis, signaling regulation, motility and invasion [33–35]. Moreover, Rab5/EEA1 vesicles provide faster and direct recycling than through recycling endosomes (1–2 vs 15–20 min) [36]. We found that Nodal is packed in EEA1/Rab5-positive vesicles in GBMsc. A possible consequence of the connection between Nodal and the endocytic pathway through Rab5/EEA1 vesicles is a higher rate of turnover of endocytated Nodal back to the membrane, maintaining Nodal signaling in the GBMsc itself, keeping in this way, high levels of Nodal in the extracellular media. This fact would contribute to keep cells in a more undifferentiated state. In fact, previous works have show that Nodal inhibition forces pluripotent embryonic stem cells into a differentiation pathway [37].

On the other hand, Rab7-positive vesicles are a key factor for enzymatic regulation and internalization of membrane surface proteins [38]. Our data show that mdGBM cells mostly presented Nodal co-localization with Rab7 and Rab11. This result indicates that these cells actively internalize Nodal and direct it for degradation or for recycling endosomes. The subsequent lower rate of Nodal addressed to the extracellular media could contribute to gradually downregulate its signaling in mdGBM cells. Altogether, our current results suggest that the endocytic pathway plays a key role for Nodal availability regulation during GBM differentiation.

We also have observed a change in Nodal intracellular distribution that was dependent upon the degree of cellular differentiation. GBMsc displayed a vesicle-like pattern of Nodal staining symmetrically distributed in the cytoplasm (Fig. 1a, b, arrow). On the other hand, mdGBM cells stained for Nodal in a more asymmetric and perinuclear fashion (Fig. 1c). We speculate that these changes directly result from the different vesicles that carry Nodal in the two cell types. EEA1/Rab5-positive vesicles would maintain Nodal closer to the membrane in GBMsc, whereas Rab7-positive vesicles would bring Nodal closer to the perinuclear region. As a consequence, GBMsc may present a higher rate of Nodal maintaining its signaling levels in the GBMsc itself.

Our immunostaining for OB1 stem cells showed that Nodal protein staining decreased as OB1 stem cells undergone differentiation (Fig. 2b, d). Our results clearly show that Nestin-positive cells are Nodal negative only when morphologically spread out, an additional indicator of differentiation. Conversely, U87MG cells forced to acquire a more undifferentiated morphology, shifted back to a symmetric pattern of Nodal cytoplasmic distribution (Additional file 4: Figure S4). These results indicate that Nodal expression heavily depends on cellular differentiation status, and we may speculate that Nodal is progressively downregulated during tumor development. In accordance, previous studies show that the TGF-β/Activin/Nodal branch is necessary to keep the pluripotent and undifferentiated state of human embryonic stem cells [5, 39]. Altogether, our results support the notion that the cancer stem cell presents a similar behavior to the embryonic stem cell in terms of Nodal function. Despite the tight correlation between Nodal signaling and the stem cell phenotype, we still noticed the presence of Nodal in mdGBM cells. We speculate that tumor cells, although they might be in a differentiation pathway, never fully acquire a final, normal differentiated phenotype—due to the innumerous transformations they have incurred. Furthermore, the similar pattern, presented by the primary culture GBM011, to the behavior of well established cell lines suggests that Nodal protein can display an innovative marker on primary culture and biopsies analysis. However, tumor heterogeneity, especially in GBM, consists in a possible limitation for the accuracy of this approach. It would be necessary to not exclude the possibility of the subpopulation taken in the sample be precisely the more differentiated subpopulation. Thus, multiple markers would consist on a foremost solution. Still, since Nodal has been correlated to cancer aggressiveness and resistance [6, 8, 10, 11], its verification in such samples would indicate the differentiation state and, consequently, improve prognosis.

Taken together, our results indicate for the first time that Nodal is consistently involved in GBM differentiation, since it is highly expressed in GBMsc and downregulated in mdGBM cells. Dedifferentiated GBM cells upregulate Nodal further supporting this hypothesis. This dynamics displayed by Nodal is tightly connected to the endocytic pathway in which Nodal is inserted. Our results also shed light on a new approach to evaluate GBM differentiation by analyzing Nodal protein subcellular distribution, shedding light on the molecular pathways that might emerge as putative targets for GBM therapy.

## Additional files

10.1186/s12935-016-0324-3 Nodal immunostaining changes along the development of the oncospheres. (a) Small sphere showing only cells that with a symmetrical Nodal distribution in the cytoplasm. (b) Large sphere comprised by cells with distinct Nodal distributions. (c–e) Nodal immunostaining (red) in OB1 stem cells placed at the top of oncospheres is localized to the cell membrane (green, phalloidin, asterisk). (f–h) Cells located at the lateral edge of oncospheres harboring different heights presented Nodal immunostaining symmetrically distributed in the cytoplasm of cells. (i–l) Cells directly attached to the substrate also presented a symmetrical distribution of Nodal. (e, h, k, l) Optical slices projected on the YZ axis showing a virtual reconstruction of the oncosphere.
10.1186/s12935-016-0324-3 Nodal protein is asymmetrically distributed in the cytoplasm of prostate cancer cells DU145. (a) Nodal immunostaining (red) is asymmetrically distributed in the perinuclear region of prostate cancer cells (DU145; arrow head). Nodal negative cell is highlighted by the asterisk. (b) Confocal image merging the bright field and Nodal immunostaining (red). (c) Nodal positive cells showing a perinuclear immunostaining (red; arrow head). (d) Confocal image merging the bright field and Nodal immunostaining. (e) Zoom of the immunopositive cell shown in d. N = nucleus.
10.1186/s12935-016-0324-3 Differentiation status validation through Nanog verification. Relative expression of Nanog in OB1, differentiated OB1, U87MG and dedifferentiated U87MG cells (quantification of average across three separate experiments). Nanog protein normalization through Actin immunoblotting. Data are mean ± SD. ****P* < 0.001 by unpaired t test, n = 3).
10.1186/s12935-016-0324-3 Nodal asymmetric cytoplasmic distribution shifts to a symmetric distribution in dedifferentiated U87MG cells. (a) In U87MG cells, Nodal immunostaining was found around the nucleus (arrows). (b) U87MG cells present Nodal immunostaining (red) symmetrically distributed in the cytoplasm of 2 different cells (asterisks). (c, d) Nodal was found symmetrically distributed in the cytoplasm of two different U87MG cells (single and double asterisk). (e) Quantification of intracellular Nodal protein in U87MG cells and upon dedifferentiation by Western blot. Nodal protein levels upregulate in 50 % upon differentiation (quantification of average across three separate experiments. Nodal protein normalization through α-Tubulin immunoblotting. Data are mean ± SD. ****P* < 0.001 by unpaired t test, n = 3).
10.1186/s12935-016-0324-3 Nodal protein co-localizes with different endosomal vesicles depending on the dedifferentiation status of GBM cell lines and GBM primary cultures. Representative images of Nodal immunostaining with endosomal markers. In U87MG-O cells, Nodal co-localized with both early (EEA1 and Rab5) and late (Rab7 and Rab11) endosomes. In contrast, in U87MG cells, Nodal immunostaining was mostly co-localized with late (Rab7 and Rab11) endosomal vesicles.
10.1186/s12935-016-0324-3 Nodal mostly co-localizes to Rab7 and Rab11 in more differentiated GBM cells and in primary cultures. (a) Pearson’s coefficient of relative amount of co-localization of endosomal markers/Nodal in U87MG-O and U87MG cell cultures (quantification of average across three separate fields, each containing an average of three to four spheroids—OB1 cells—or 20 to 30 cells—differentiated OB1 cells). (b) Pearson’s coefficient of relative amount of co-localization of endosomal markers/Nodal in GBM011 cells (quantification of average across three separate fields, each containing an average of 20–30 cells). Data means are ± SD. ****P* < 0.001 by two-way ANOVA for repeated measures followed by Tukey’s test for correction of the *P* value.

## Abbreviations

GBM: glioblastoma
TGF-β: transforming growth factor-β
GBMsc: GBM stem cells
mdGBM: more differentiated GBM
UFRJ: Universidade Federal do Rio de Janeiro
FBS: fetal serum bovine
CM: conditioned medium
SD: standard deviation
