# Supplementary figures and images for: The availability of the embryonic TGF-β protein Nodal is dynamically regulated during glioblastoma multiforme tumorigenesis
Source: Cancer Cell Int. 2016 Jun 17;16:46. doi: 10.1186/s12935-016-0324-3 (PMC4912793; doi:10.1186/s12935-016-0324-3)

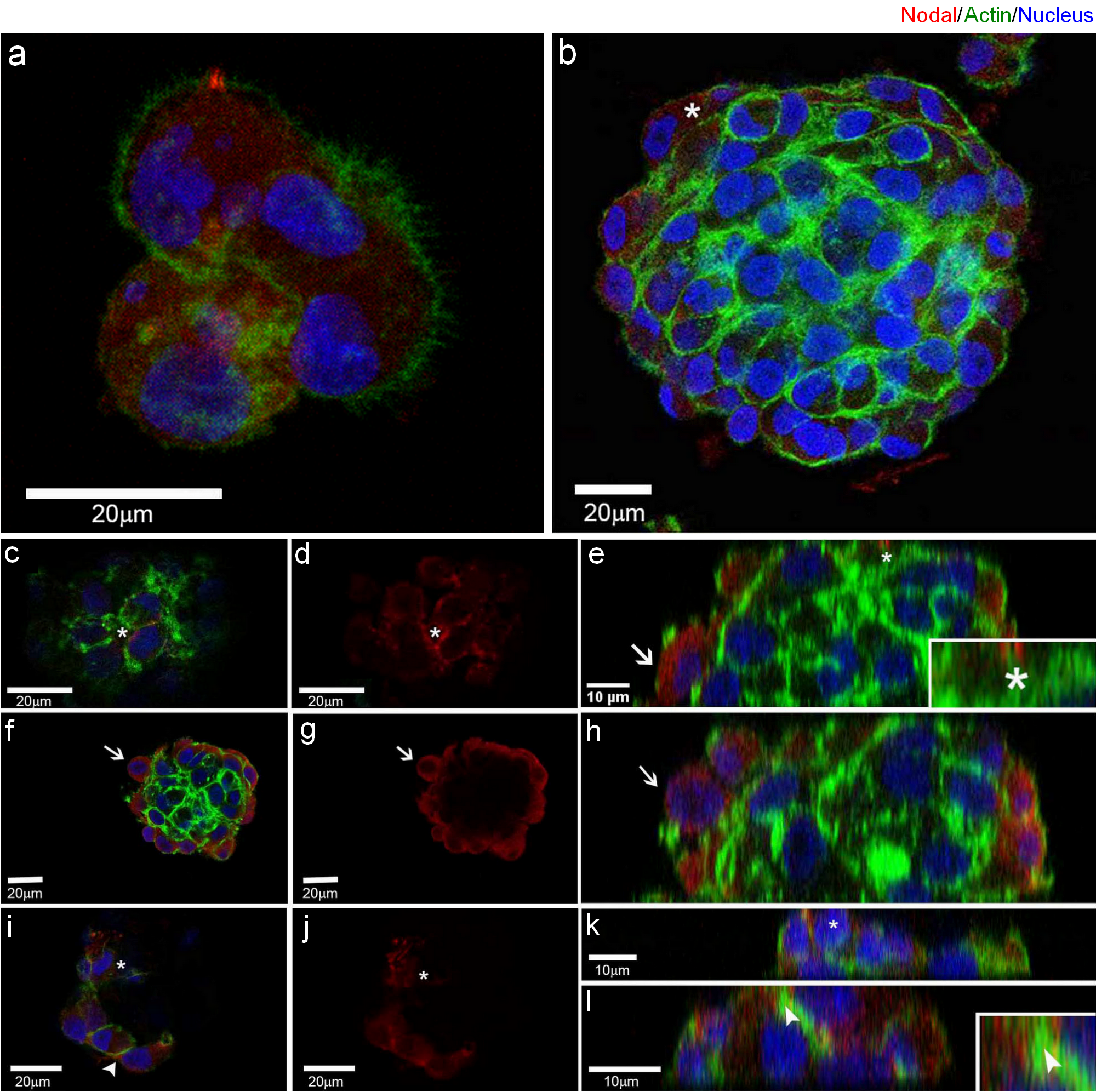

Supplement: Supplementary file 1 — 10.1186/s12935-016-0324-3 Nodal immunostaining changes along the development of the oncospheres. (a) Small sphere showing only cells that with a symmetrical Nodal distribution in the cytoplasm. (b) Large sphere comprised by cells with distinct Nodal distributions. (c–e) Nodal immunostaining (red) in OB1 stem cells placed at the top of oncospheres is localized to the cell membrane (green, phalloidin, asterisk). (f–h) Cells located at the lateral edge of oncospheres harboring different heights presented Nodal immunostaining symmetrically distributed in the cytoplasm of cells. (i–l) Cells directly attached to the substrate also presented a symmetrical distribution of Nodal. (e, h, k, l) Optical slices projected on the YZ axis showing a virtual reconstruction of the oncosphere. [file 12935_2016_324_MOESM1_ESM.tif]

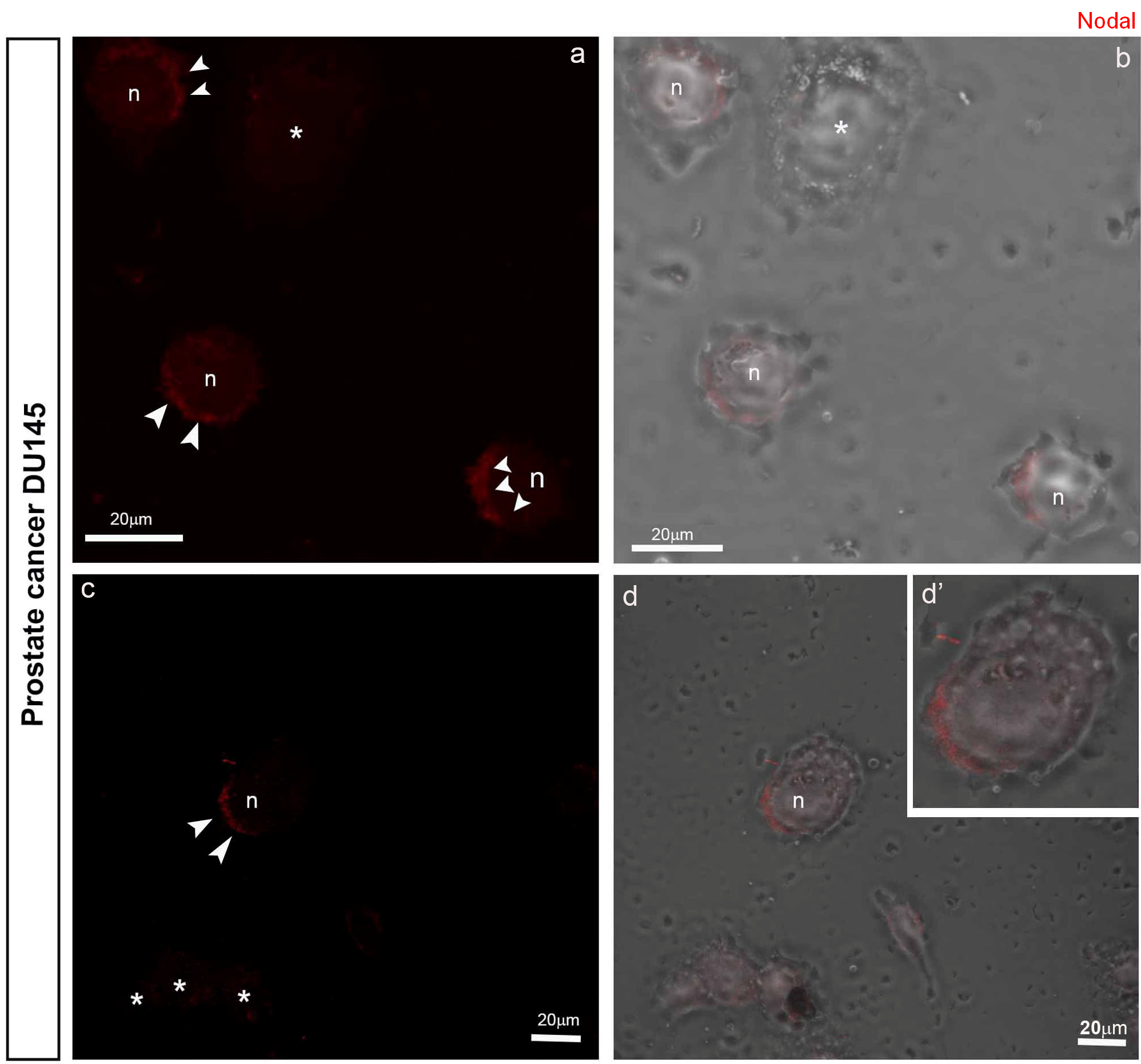

Supplement: Supplementary file 2 — 10.1186/s12935-016-0324-3 Nodal protein is asymmetrically distributed in the cytoplasm of prostate cancer cells DU145. (a) Nodal immunostaining (red) is asymmetrically distributed in the perinuclear region of prostate cancer cells (DU145; arrow head). Nodal negative cell is highlighted by the asterisk. (b) Confocal image merging the bright field and Nodal immunostaining (red). (c) Nodal positive cells showing a perinuclear immunostaining (red; arrow head). (d) Confocal image merging the bright field and Nodal immunostaining. (e) Zoom of the immunopositive cell shown in d. N = nucleus. [file 12935_2016_324_MOESM2_ESM.tif]

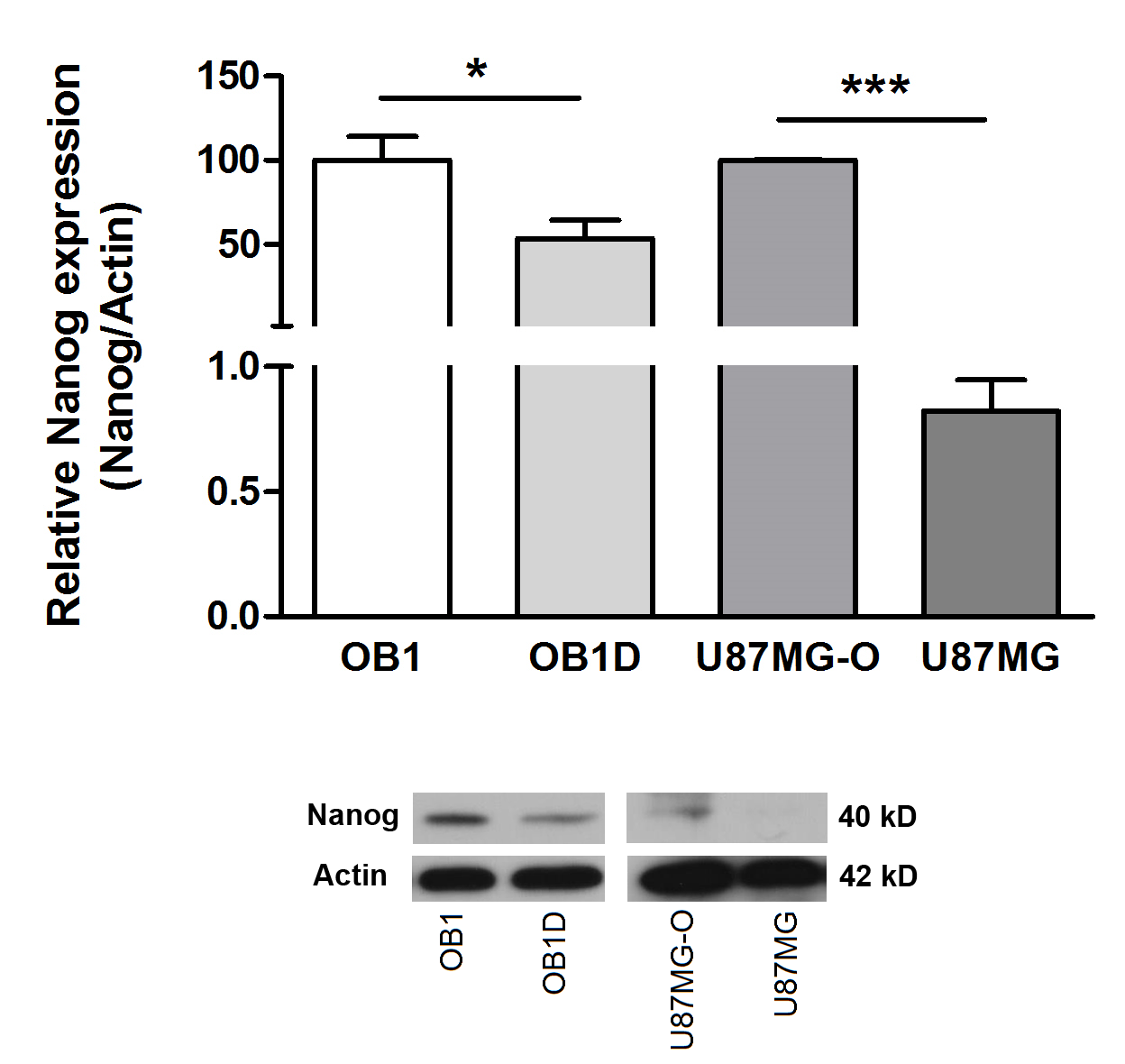

Supplement: Supplementary file 3 — 10.1186/s12935-016-0324-3 Differentiation status validation through Nanog verification. Relative expression of Nanog in OB1, differentiated OB1, U87MG and dedifferentiated U87MG cells (quantification of average across three separate experiments). Nanog protein normalization through Actin immunoblotting. Data are mean ± SD. ***P < 0.001 by unpaired t test, n = 3). [file 12935_2016_324_MOESM3_ESM.tif]

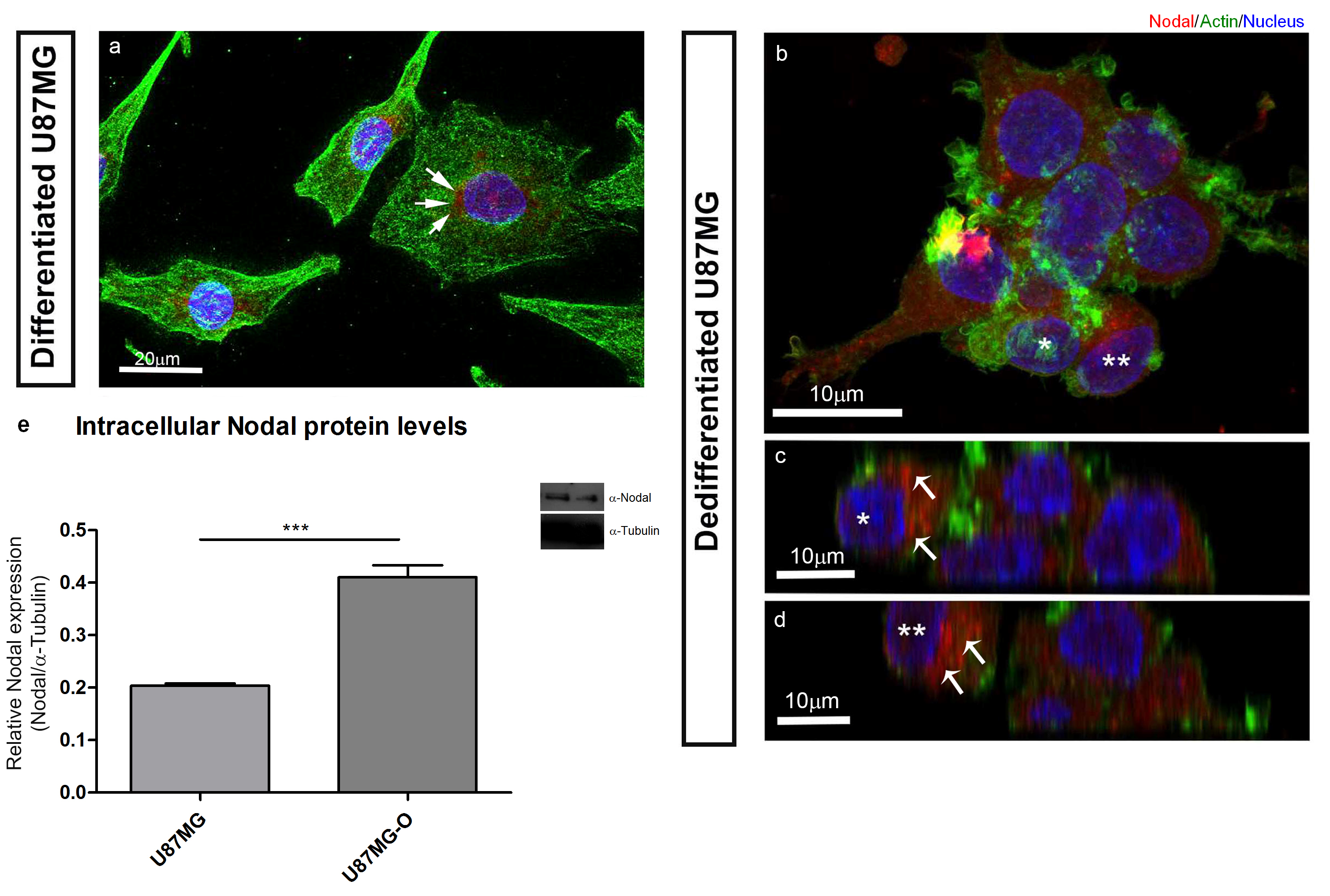

Supplement: Supplementary file 4 — 10.1186/s12935-016-0324-3 Nodal asymmetric cytoplasmic distribution shifts to a symmetric distribution in dedifferentiated U87MG cells. (a) In U87MG cells, Nodal immunostaining was found around the nucleus (arrows). (b) U87MG cells present Nodal immunostaining (red) symmetrically distributed in the cytoplasm of 2 different cells (asterisks). (c, d) Nodal was found symmetrically distributed in the cytoplasm of two different U87MG cells (single and double asterisk). (e) Quantification of intracellular Nodal protein in U87MG cells and upon dedifferentiation by Western blot. Nodal protein levels upregulate in 50 % upon differentiation (quantification of average across three separate experiments. Nodal protein normalization through α-Tubulin immunoblotting. Data are mean ± SD. ***P < 0.001 by unpaired t test, n = 3). [file 12935_2016_324_MOESM4_ESM.tif]

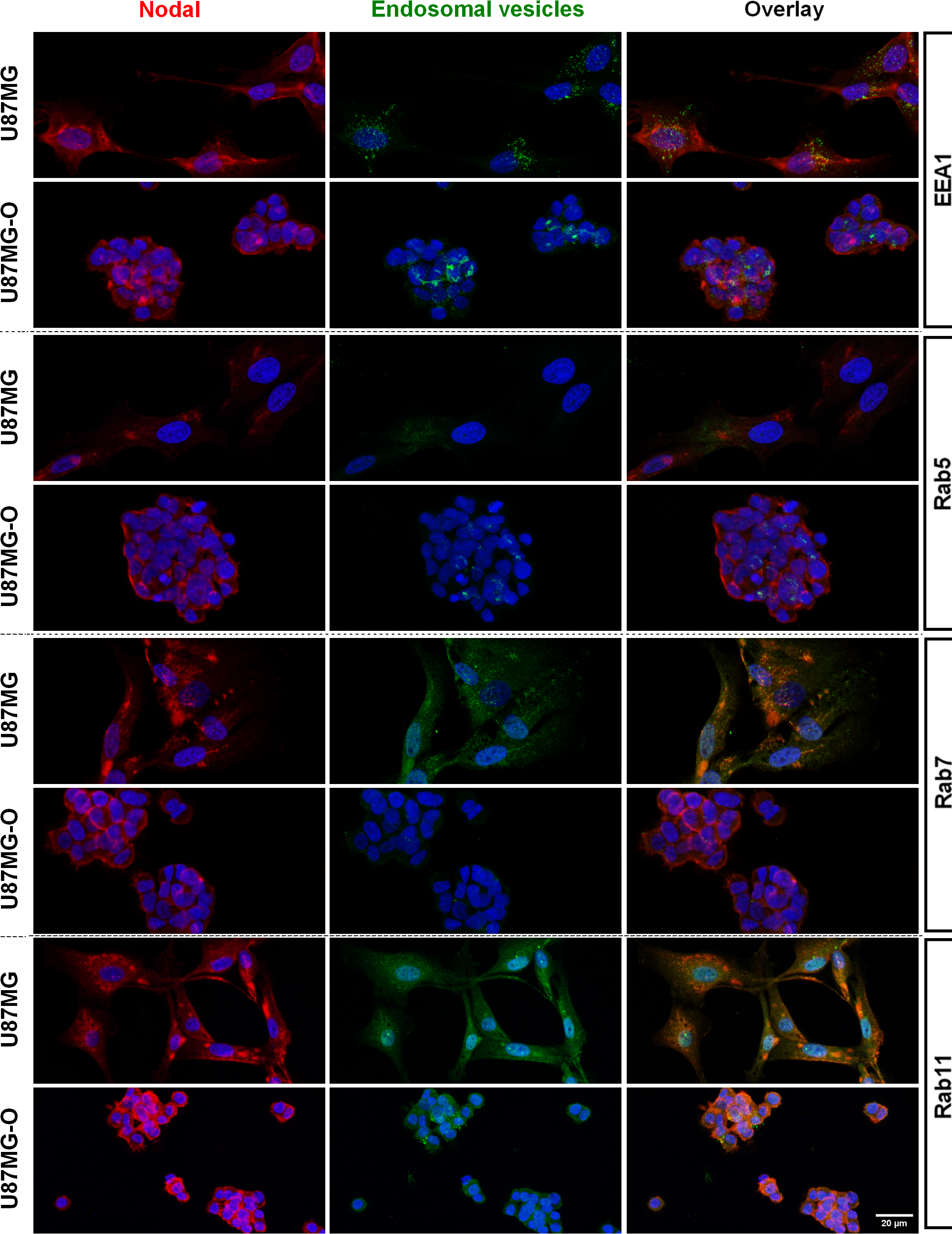

Supplement: Supplementary file 5 — 10.1186/s12935-016-0324-3 Nodal protein co-localizes with different endosomal vesicles depending on the dedifferentiation status of GBM cell lines and GBM primary cultures. Representative images of Nodal immunostaining with endosomal markers. In U87MG-O cells, Nodal co-localized with both early (EEA1 and Rab5) and late (Rab7 and Rab11) endosomes. In contrast, in U87MG cells, Nodal immunostaining was mostly co-localized with late (Rab7 and Rab11) endosomal vesicles. [file 12935_2016_324_MOESM5_ESM.tif]

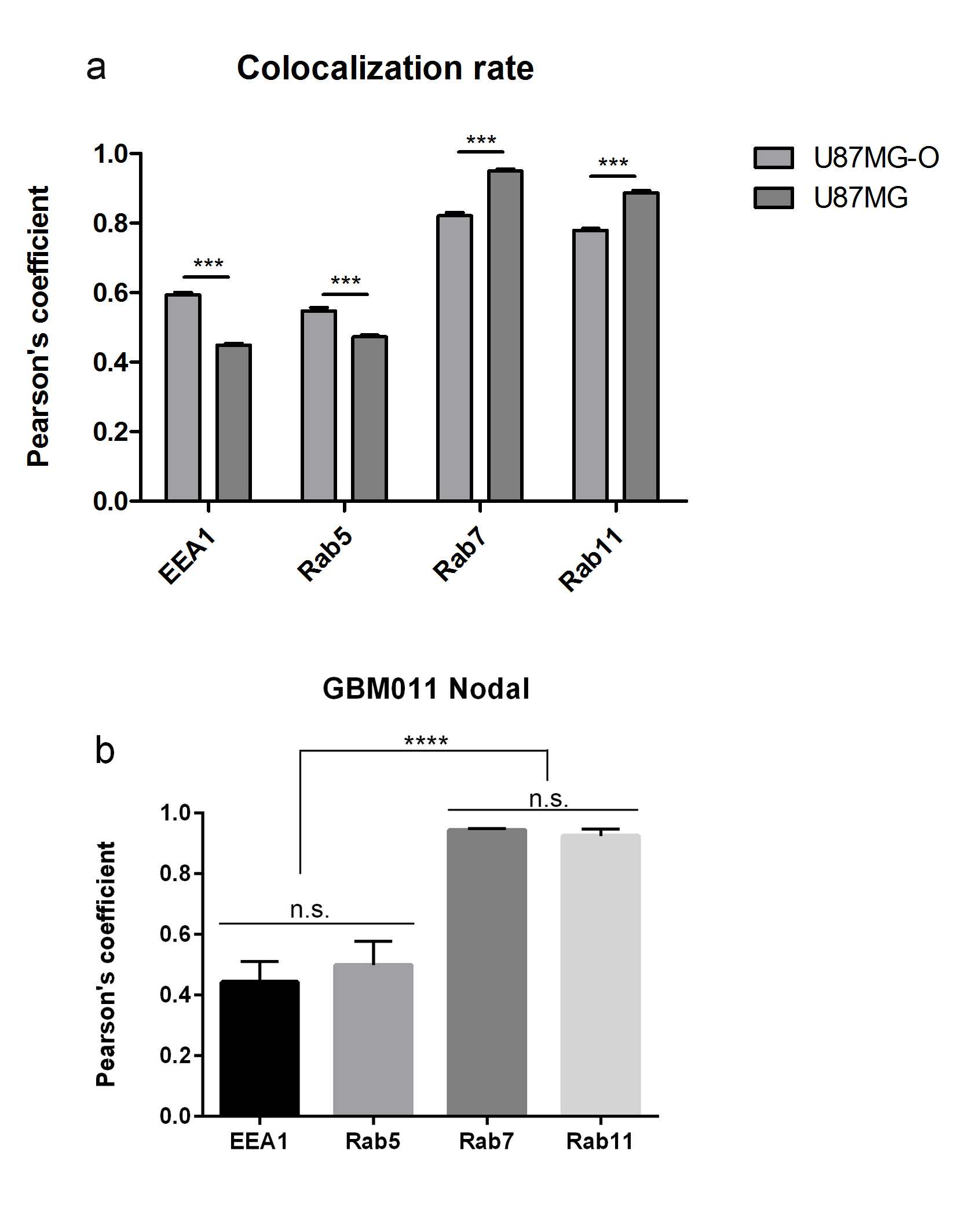

Supplement: Supplementary file 6 — 10.1186/s12935-016-0324-3 Nodal mostly co-localizes to Rab7 and Rab11 in more differentiated GBM cells and in primary cultures. (a) Pearson’s coefficient of relative amount of co-localization of endosomal markers/Nodal in U87MG-O and U87MG cell cultures (quantification of average across three separate fields, each containing an average of three to four spheroids—OB1 cells—or 20 to 30 cells—differentiated OB1 cells). (b) Pearson’s coefficient of relative amount of co-localization of endosomal markers/Nodal in GBM011 cells (quantification of average across three separate fields, each containing an average of 20–30 cells). Data means are ± SD. ***P < 0.001 by two-way ANOVA for repeated measures followed by Tukey’s test for correction of the P value. [file 12935_2016_324_MOESM6_ESM.tif]
